# Mask use in public places in Maputo City, Mozambique: Cross-sectional survey

**DOI:** 10.1371/journal.pone.0288957

**Published:** 2023-08-02

**Authors:** Dionísia Balate, Gerson Afai, Faiza Sallé, Timóteo Simone, Cynthia Semá Baltazar, Rose Zulliger, Érika Valeska Rossetto

**Affiliations:** 1 Field Epidemiology Training Program, National Institute of Health, Maputo, Mozambique; 2 Survey and Surveillance Department, National Institute of Health, Maputo, Mozambique; 3 US Centers for Disease Control and Prevention, Maputo, Mozambique; 4 U.S. President’s Malaria Initiative, US Centers for Disease Control and Prevention, Maputo, Mozambique; 5 MassGenics assigned to US Centers for Disease Control and Prevention, Maputo, Mozambique; Eduardo Mondlane University: Universidade Eduardo Mondlane, MOZAMBIQUE

## Abstract

**Introduction:**

The use of face masks is one of the preventive measures that Mozambique adopted in order to limit the spread of COVID-19. A study carried out from May 25 to June 6, 2020 found that although many wore masks, incorrect use was observed in 27.5% of the population observed. This data collection aimed to measure the degree of mask use compliance during a more protracted, higher second wave of transmission.

**Methodology:**

A cross-sectional study was conducted in the City of Maputo from 19 to 28 October 2020 through direct observation of mask use of all individuals present in markets, supermarkets and bus terminals. The data were collected using mobile phones with the Open Data Kit Collect (ODK) data collection program. Sociodemographic characteristics, mask use, and type of mask used were documented. Factors associated with incorrect mask use were evaluated considering sex, age, observation period and location.

**Results:**

A total of 49,404 individuals were observed, of whom 24,977(50.6%) were male, 46,484 (94.1%) were adults and 17,549 (35.5%) were observed in the markets. An observed 41,786 (84.6%) wore a mask, of whom 33,851 (81.0%) used it correctly. Not covering the mouth and nose was common; observed in 4,649 (58.5%) of those using incorrectly. Of different types of masks, fabric masks were most often used incorrectly 7,225 (21.4%). The factors associated with incorrect mask use were female gender (OR = 1.2 [1.1–1.3], p <0.001), observation in peri-urban versus urban areas (OR = 1.9 [1.8–2.1], p <0.001) and observation during the afternoon (OR = 1.5 [1.5–1.6], p <0. 001).

**Conclusion:**

A high proportion of observed individuals wore a mask in the context of prevention of COVID-19, however some non-use and incorrect use persists. Intensified public awareness of the correct use of the mask is recommended, especially in peri-urban areas and at the end of the day.

## Background

On December 31, 2019, China reported to the World Health Organization (WHO) a cluster of pneumonia cases of unknown etiology in Wuhan city, Hubei province [1]. The outbreak spread quickly, with an increasing number of cases reported worldwide [2]. In February 2020, the WHO designated the disease caused by Severe Acute Respiratory Syndrome Coronavirus 2 (SARS-CoV-2) as coronavirus disease (COVID-19) [1]. More than 280,000,000 COVID-19 confirmed cases and 5,000,000 deaths worldwide were reported by the end of 2021, of which more than 700,000 confirmed cases and 150,000 deaths were reported in Africa [3].

In Mozambique, the first case of COVID-19 was registered on March 22, 2020 and by October 30 the same year there were 12,777 confirmed cases and 91 deaths registered [4]. To control the disease’s spread in Mozambique, a 30-day State of Emergency was declared on March 31, 2020. This declaration was extended for two additional months [5]. On September 7, 2020 a State of Public Disaster was declared, which remained in effect during the conduct of this study [6]. This State of Public Disaster required compliance with hand hygiene, physical distancing and the use of masks for the prevention and control of COVID-19.

According to the WHO, the use of masks is part of a comprehensive package of prevention and control measures that can limit the spread of certain viral respiratory diseases, including COVID-19 [7]. The types of masks recommended by the WHO are surgical masks, N95 masks, and non-medical or homemade masks [8]. One study conducted in the United States of America found that mandating face mask use in public was associated with a 2.0 percentage point decline in the daily COVID-19 cases growth rate 21 or more days post implementation [9]. In another large time-interrupted series study in Melbourne, has shown that the reduction of COVID-19 was about 30% depending on the assumptions about serial interval time [10].

A pilot study on mask-wearing behavior in the city of Maputo, Mozambique carried out by Eduardo Mondlane University in February 2020 found that many people (72,9%) were observed wearing masks [11]. However, among those using masks there were still individuals who did not use the masks correctly (27,1%), and that claimed the use of masks caused allergy or difficulty breathing, especially for people with respiratory problems [11]. In Mozambique, the use of masks is mandatory in all public and private transport as well as in all agglomerations of people [12].

With the State of Public Disaster decreed in the country and the start of a more protracted, higher second wave of cases, there was a need to re-assess the use of masks. The aim of the study was to evaluate the use of masks in the prevention of COVID-19, in Maputo City from October 19 to 28, 2020.

## Methodology

### Study design and location

We carried out a cross-sectional study of mask use among individuals in five community markets, six supermarkets, and six open air bus terminals in the urban and peri-urban areas of Maputo City from 19 to 28 October 2020. The community markets differ from the supermarkets in that they open-air spaces composed of individual vendors largely selling goods such as agricultural and handmade products rather than large commercial establishments.

We used a convenience, non-probabilistic sampling method. The duration of the observations for each selected site was for two consecutive hours, from 6 am to 8 am, from 9 am to 11 am, and from 3 pm to 5 pm, considering these time intervals as the most crowded during the day. Each location was observed during the three periods on the same day. Investigators standardized a work schedule to ensure complete observation by day and location. Data collection was done by Field Epidemiology Training Program residents after being trained on the study protocol.

In the places selected for the study, some people passing by the place were observed. For the markets, the observations were made according with the layout of the stands/infrastructures and for the bus terminals, the observations were made as a function of the queues. In addition, observers were placed in positions that allowed discreet observation so as to limit their influence on population movements and mask use behaviours.

For the definition of age groups, children were those who appeared to be under 18 years old, and those who appeared to be over 18 years old were categorized as adults, based on observer’s judgement.

The correct use of the mask consists of covering the mouth and nose with a mask and making sure that there is no gap between the face and the mask [13].

The data were collected through direct observation with data collection via smartphones. The Open Data Kit (ODK Collect) data collection program was used, in a form that worked offline and online. The study variables included the name and type of the observation location, observation period, age and sex of the observed individual, types of masks worn (surgical, cloth, N95 and visor/face shield worn) and how the masks were worn (correct or incorrect use). For incorrect mask use, the following options were provided: uncovered nose, uncovered mouth, uncovered nose and mouth. A mask that completely covered mouth and nostrils was considered to be used correctly.

### Data analysis

Data were analyzed using the STATA 16.1 statistical program (StataCorp LLC, College Station, Texas). Descriptive analysis was conducted by calculating frequencies, presented in tables and graphs produced in Microsoft Office Excel 2013. Unconditional multivariable logistic regression analysis was conducted to identify factors associated with the incorrect mask use to produce unweighted estimates. Incorrect mask use was considered as the dependent variable, and gender, age, study area, and period of observation were considered independent variables. P values <0.05 were considered statistically significant.

### Ethical considerations

The study protocol was approved by the National Bioethics Committee. This activity was reviewed by CDC and was conducted consistent with applicable federal law and CDC policy.

## Results

During the study period, a total of 49,404 individuals were observed; 50.6% were male, 94.1% were adults >18 years of age, and 35.5% were observed in community markets (Table 1).

**Table 1:** Distribution of people observed by age group, sex and place of observation (n = 49,404) in evaluation of mask use in public places in the context of COVID-19, 19 to 28 October 2020, Maputo City, Mozambique.

Places	Children (<18 years)		Adults (18 ≥ years)		Total
	Male	Female	Male	Female	
	n (%)	n (%)	n (%)	n (%)	
**Supermarkets**	496 (3.1)	470 (2.9)	7,351 (45.6)	7,808 (48.4)	16,125
**Community markets **	630 (3.6)	518 (3.0)	8,452 (48.2)	7,949 (45.3)	17,549
**Bus terminals**	432 (2.7)	374 (2.4)	7,636 (48.5)	7,288 (46.5)	15,730
**Total**	1,558 (3.2)	1,362 (2.8)	23,439 (47.4)	23,045 (46.6)	49,404

### Distribution of mask use

Regarding mask use, 84.6% of those observed used a mask, of whom, 50.4% were females and 49.5% were males. Mask use was lowest among children (4.8% wore a mask); 95.2% of adults wore masks. In urban areas 96.1% of people observed wore masks and 3.9% wore them in peri-urban areas. Regarding the place of observation, 36.0% of the individuals who used the mask were observed in the market, followed by the supermarket with 32.0% (Table 2).

**Table 2:** Distribution of mask use (N = 49,404) in public places, from 19 to 28 October 2020 Maputo City, Mozambique.

Characteristics		Used		Did not use		Total	
		(N = 41786)		(N = 7618)		(N = 49404)	
		N	%	n	%	n	%
	Male	20,711	49.6	4,286	56.3	24,997	50.6
	Female	21,075	50.4	3,332	43.7	24,407	49.4
**Age range**	Children (<18 years)	2,015	4.8	905	11.9	2,920	5.9
	Adults (≥ 18 years)	39,771	95.2	6,713	88.1	46,484	94.1
**Area**	Urban	40,165	96.1	6,966	91.4	47,131	95.4
	Peri-urban	1,621	3.9	652	8.6	2,273	4.6
**Place**	Supermarket	13,722	32.8	2,403	31.5	16,125	32.6
	Market	15,051	36.0	2,498	32.8	17,549	35.5
	Bus terminals	13,013	31.1	2,717	35.7	15,730	31.8

### Correct mask use

Of the 41,786 people who used masks, 33,851 (81.0%) used them correctly. Of these, 95.3% were adults, 50.6% were male, 61.7% were observed in the morning, 96.8% were located in urban areas.

The correct use of the mask was 36.3% in markets, and 32.9% in supermarkets. The fabric mask was the most used correctly with 78.9%. Of the 7,935 people who used the mask incorrectly, the most frequent misuse was not covering the mouth and nose, observed in 58.6% of pelople (Table 3).

**Table 3:** Proper mask use among mask wearers (N = 41,786) observed from 19 to 28 October 2020, Maputo City, Mozambique.

Characteristics		Total N = 41,786	Mode of use of mask			
			Correct		Incorrect	
			n = 33,851	%	n = 7,935	%
**Age**	Children (<18 years)	2,015	1,589	4.7	426	5.4
	Adults (≥ 18 years)	39,771	32,262	95.3	7,509	94.6
**Gender**	Male	20,711	17,123	50.6	3,588	45.2
	Female	21,075	16,728	49.4	4,347	54.8
**Time of observation**	Morning	24,868	20,902	61.7	3,966	50.0
	Afternoon	16,918	12,949	38.3	3,969	50.0
**Area**	Urban	40,165	32,779	96.8	7,386	93.1
	Peri-urban	1,621	1,072	3.2	549	6.9
**Place**	Supermarket	13,722	11,143	32.9	2,579	7.0
	Market	15,051	12,282	36.3	2,769	0.9
	Bus terminal	13,013	10,426	30.8	2,587	91.4
**Mask type**	Surgical	6,239	5,684	16.8	555	7.0
	N95	1,150	1,081	3.2	69	0.9
	Cloth	33,965	26,710	78.9	7,255	91.4
	Visor/face shield	432	376	1.1	56	0.7
**Form of incorrect use**	Nose not covered	-	-	-	3,254	41.0
	Mouth not covered	-	-	-	32	0.4
	Uncovered mouth and nose	-	-	-	4,649	58.6

### Factors associated with the incorrect use of masks

In multivariable regression analysis, the factors significantly associated with incorrect mask use were being female (adjusted Odds Ratio, aOR = 1.2 [1.1–1.3] p <0.001), being observed in peri-urban versus urban areas (aOR = 1.9 [1.8–2.1] p <0.001), and afternoon versus morning observation period/time (aOR = 1.5 [1.5–1.6] p <0.001) (Table 4).

**Table 4:** Factors associated with the incorrect use of the masks in public places, Maputo City, 19 to 28 October 2020.

Characteristics		N = 7,935	%	Univariate analysis		Multivariable analysis	
				OR (CI 95%)	*p*-value	aOR (CI 95%)	*p*-value
**Gender**	Male	3,588	45.2	Ref	-	-	-
	Female	4,347	54.8	1.2 (1.2–1.3)	<0,001	1.2 (1.1–1.3)	<0.001
**Age**	Children	426	5.4	1.2 (1.0–1.3)	0.012	0.9 (0.7–1.3)	0.949
	Adult	7,509	94.6	Ref	-	-	-
**Area**	Urban	7,386	93.1	Ref	-	-	-
	Peri-urban	549	6.9	2.3 (2.0–2.5)	<0.001	1.9 (1.8–2.1)	<0.001
**Period**	Morning	3,966	50	Ref	-	-	-
	Afternoon	3,969	50	1.6 (1.5–1.7)	<0.001	1.5 (1.5–1.6)	<0.001

aOR: adjusted *Odds Ratio*; CI: Confidence Interval; Ref: Reference Category

## Discussion

The use of face masks is an effective means to prevent and contain the spread of COVID-19 [14]. This study evaluated the use of face masks in the capital of Mozambique during the COVID-19 pandemic in October of 2020. The majority, 84.6%, of the observed individuals used masks. Similar results were found in a pilot study on mask-wearing behavior in the city of Maputo, where 72% of those observed wore a mask and another study on Adherence to COVID-19 Preventive Measures in Mozambique, where adherence rates were 90% [11, 15].

This study included observations in different locations that provide valuable evidence in public areas with high population density that might represent high transmission risk. Most of those observed were in the markets where the greater concentration and flow of people could create a favorable environment for the transmission of COVID-19, indicating the importance of preventive measures in these settings.

Masks provide protection to the user when used correctly [15, 16]. Although most people observed used their masks correctly, there were also individuals who used them incorrectly, most frequently leaving their nose and mouth uncovered. This fact may be related to the discomfort associated with the length of use of the mask as other studies have revealed that 22.0% of mask removals were due to discomfort and prolonged use, for example sweating or difficulty breathing [16, 17].

The odds of incorrect use were significantly higher among women, in peri-urban areas and during the afternoon period. Women were slightly more likely to use a mask than men, but their significantly poorer likelihood of correct use is indicative of a need for more targeted messaging on how to correctly use masks. Those in peri-urban areas were less likely to use a mask at all and were significantly less likely to have correct mask use which highlights the need for broadening the scope of communication campaigns outside of urban areas where mask use may be worse.

Cloth masks were the type of masks used most frequently by observed individuals. This may be due to this type of masks’ availability, low cost, and possibility of reuse after washing. The use of multi-layer cloth masks is a simple, economical and sustainable alternative to surgical masks as a means of source control for general community use, as is strongly supported by the evidence [18]. Fabric masks may be less fitted than N95 masks and, in turn, have less impact on breathing [19, 20]. Additionally, there have been arguments that for the population to have access to a mask, it was necessary that it be a low-cost one [21], underscoring the value of reusable, inexpensive cloth masks such as those frequently observed in this study. However, these masks are not without some concern as at least one randomized trial found a higher rate of respiratory infections in the group using a cloth mask versus those with surgical masks [22]. Nonetheless, a follow-up study found cloth masks provided equal protection to medical masks for healthcare workers when laundered by the hospital. Additionally, research carried out in Nepal showed that the effectiveness of fabric masks is reduced by 20.0% after the fourth washing and drying Optical microscopic study [20]. Despite these potential concerns related to cloth masks, the lower impact on breathing, lower costs, and higher availability may have contributed to the fact that these were the most frequently documented masks in this study. As such, communication efforts might be most effective if they use imagery of cloth masks and focus on promotion of the correct and consistent use of such masks.

While this study provides valuable insights into frequency and type of mask use in Maputo City, it has some important limitations. These include limitations based on the observational methods which limited assessment of the quality or materials used for the masks, the required estimations of ages and the availability of data that could help illustrate the underlying reasons for any statistically significant differences in correct mask use between groups. Additionally, during data collection a subgroup for the elderly was not categorized, limiting our insight into how mask use may vary in this more vulnerable population.

This study did not include rural areas and other congregated environments and it utilized convenience sampling to help provide data on trends in mask use over time. Each person was only observed over a short time period, so we could not measure consistency of mask use at individual-level. Because the period of observation was brief, the unweighted estimates from this study provide a snapshot of use during the specific regulatory and transmission context that continues to rapidly evolve, underscoring the value of repeating this exercise over time and in more geographic settings.

## Conclusions and recommendations

The public observed in urban and peri-urban Maputo City, Mozambique used masks to prevent COVID-19 transmission, however, some individuals used them incorrectly. Incorrect use of masks was associated with being female, living in a peri-urban area and being observed in the afternoon. Intensified public awareness of the correct use of face masks is recommended, especially in peri-urban areas and in the afternoon. Additionally, there is value in continuous monitoring and enforcement of compliance with the rules for wearing masks for the prevention of COVID-19 in public places.

## Supporting information

S1 Checklist STROBE statement—checklist of items included in reports. (DOCX)
